# Ovarian Mass Causing Paradoxical MI and Leg Ischaemia

**DOI:** 10.1155/2012/702509

**Published:** 2012-10-10

**Authors:** K. J. Griffin, M. A. Bailey, J. P. Greenwood, L. Barker, T. Nicholson, D. J. A. Scott

**Affiliations:** ^1^Leeds Vascular Institute, Leeds General Infirmary, Great George Street, Leeds LS1 3EX, UK; ^2^Yorkshire Heart Centre, Leeds General Infirmary, Great George Street, Leeds LS1 3EX, UK; ^3^Department of Pathology, Leeds Teaching Hospitals Trust, Leeds LS1 3EX, UK; ^4^Department of Interventional Radiology, Leeds Teaching Hospitals Trust, Leeds LS1 3EX, UK

## Abstract

Paradoxical embolus through a patent foramen ovale is a well-reported phenomenon. Clinical consequences include stroke, intestinal infarction, lower limb ischaemia, and even acute myocardial infarction (MI), via embolisation to the coronary arteries. We present a case of acute MI, cardiogenic shock, and cardiac arrest caused not by this mechanism, but by embolisation of thrombotic material to the aortic root with transient complete occlusion of the left main stem (LMS) coronary artery. During percutaneous coronary intervention to treat this occlusion the thrombus became lodged at the aortic bifurcation causing lower limb ischaemia. Despite successful treatment of this via bilateral groin exploration and thromboembolectomy the patient became increasingly acidotic and an abdominal and pelvic CT scan was performed. This revealed the source of the thrombus to be the patient's congested and compressed pelvic veins which were the result of a large, previously undiagnosed ovarian malignancy with metastatic spread. Although very unusual we feel this case highlights an important differential in the diagnosis of anterolateral MI and images similar to those presented here are previously unreported in the literature.

## 1. Introduction

Paradoxical embolus through a patent foramen ovale is a well-reported phenomenon. Clinical consequences include stroke, intestinal infarction, lower limb ischaemia, and even acute myocardial infarction (MI), via embolisation to the coronary arteries. We present a case of acute MI, cardiogenic shock, and cardiac arrest caused not by this mechanism, but by embolisation of thrombotic material to the aortic root with transient complete occlusion of the left main stem (LMS) coronary artery.

## 2. Case Presentation

**Figure 1:**
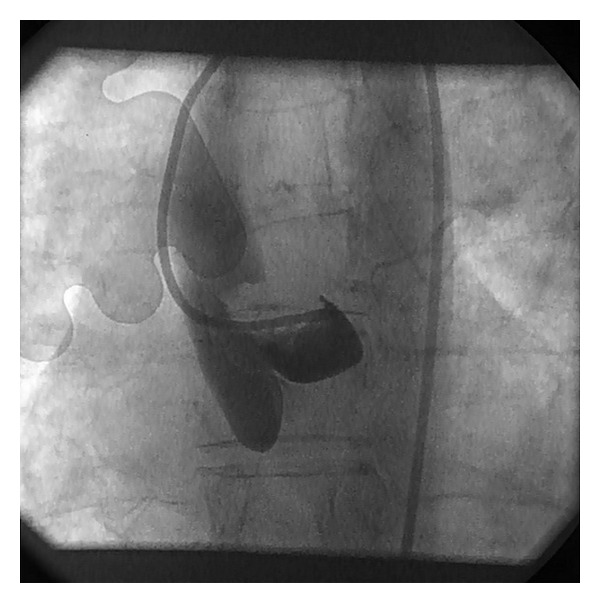
Initial aortogram showing aortic root mass.

A 57-year-old woman presented acutely to the primary percutaneous coronary intervention (PCI) service at our institution with a history of indigestion-type symptoms for several hours followed by acute shortness of breath, back, and chest pain. She was previously fit and well with no personal or family history of cardiac disease. Her electrocardiogram (ECG) showed typical changes of anterolateral ST elevation myocardial infarction (STEMI) and she was transferred immediately to the catheterisation lab. The patient was haemodynamically unstable with signs of cardiogenic shock; femoral arterial and venous sheaths were inserted on arrival. Initial coronary angiography revealed a large irregular mass in the aortic root (Figure 1) causing complete occlusion of the left main stem coronary artery with no left-sided coronary perfusion. A standard intracoronary wire was rapidly inserted into the left anterior descending artery with the plan to “stent” the LMS ostium and restore antegrade blood flow, however, at this stage the patient arrested and cardiopulmonary resuscitation was initiated and the patient intubated and ventilated. 

**Figure 2:**
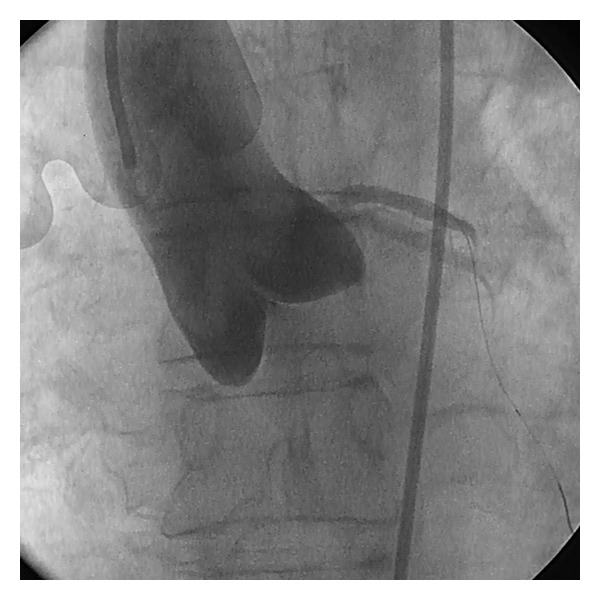
Repeat aortogram showing no aortic mass and improved coronary perfusion with bare metal wire *in situ* in the left coronary artery.

**Figure 3:**
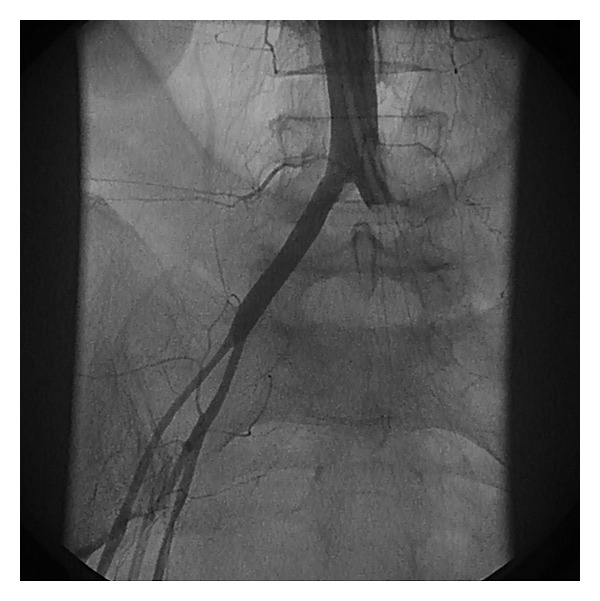
Angiogram showing complete occlusion of the left common iliac artery with no distal flow and collapsed right external iliac systems consistent with hypotension.

The differential diagnosis at this stage was between atrial myxoma (found rarely on the aortic valve), aortic root thrombus, or possible dissection. On return of cardiac output (within minutes) a repeat aortogram via the coronary guide-catheter was performed (Figure 2). Surprisingly this imaging showed no evidence of an aortic root mass and the left coronary arteries were both widely patent (TIM I II to III flow) other than a clearly defined distal occlusion of the obtuse marginal (OM_1_) branch consistent with microembolisation. These findings were confirmed on transoesophageal echo (TOE) which also demonstrated anterior wall akinesis (as expected) and a patent foramen ovale with a small left-to-right shunt. However, an angiogram of the distal aorta revealed occlusion of the left common iliac artery with compromised lower limb circulation evident both angiographically (Figure 3) and clinically.

At this stage a vascular surgery opinion was sought and the decision made to perform urgent open groin exploration with thromboembolectomies bilaterally in the cardiac cath-lab. The patient remained critically unwell and required an intra-aortic balloon pump as well as increasing inotropic support to augment cardiac output. Successful catheter trawl proximally and distally restored lower limb perfusion and embolic material was sent for emergent histology, whilst four-compartment calf fasciotomies were performed to prevent the development of subsequent compartment syndrome. Despite this acute management the patient's clinical state continued to deteriorate with worsening metabolic acidosis, rising lactate levels, and falling blood glucose.

**Figure 4:**
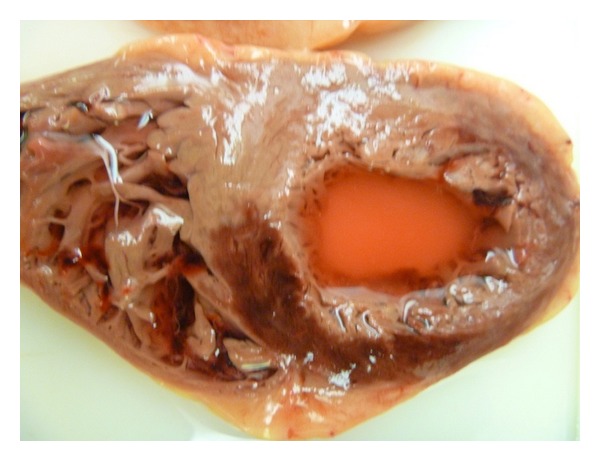
Postmortem photographs illustrating a large anterolateral myocardial infarction.

**Figure 5:**
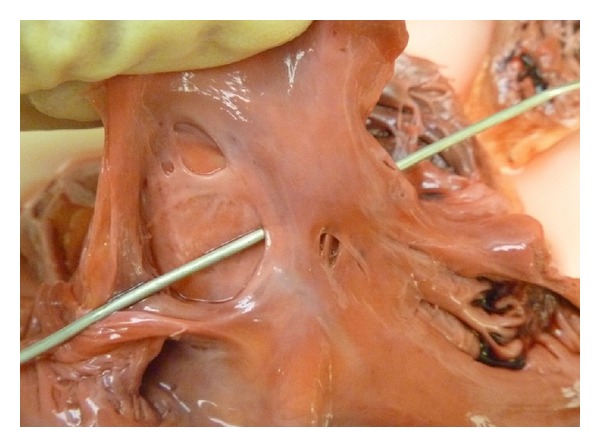
Postmortem photographs illustrating the presence of a patent foramen ovale.

An urgent CT scan was undertaken to exclude significant mesenteric ischaemia, however, this revealed the presence of a huge ovarian tumour with pelvic venous congestion, thrombus, and evidence of widespread metastatic spread or “omental cake”. There was no evidence of bowel, renal or liver ischaemia however the patient continued to deteriorate and ITU support was sadly withdrawn after consultation with the patients' family and clinical team. Blood tests taken prior to deterioration did not reveal any evidence of thrombophilia although routine clotting parameters were deranged due to the patient's poor clinical state. A postmortem examination reinforced the clinical and radiological findings (Figures 4 and 5) and histology confirmed the ovarian malignancy and that the embolic material consisted of simple thrombus only.

## 3. Discussion

Although a sad and thankfully rare case, we feel this paper illustrates an important differential in the cause of acute myocardial infarction. Paradoxical embolus through a patent foramen ovale is a well-reported phenomenon. Clinical consequences include stroke, intestinal, or renal infarction, and lower limb ischaemia. Paradoxical embolism to the coronary arteries has been previously proposed as a cause of MI [1] and both cardiac tumours [2, 3] and floating thrombus (due to Protein C deficiency) [4] have been documented as causing coronary artery occlusion in single case reports. 

To our knowledge this is the first report of a venous thromboembolism causing complete coronary artery occlusion and subsequent iliac obstruction after entering the arterial circulation via PFO. As such this is a novel condition which highlights many learning points in terms of diagnosis and management, not least the importance of multidisciplinary team working between surgical, radiological, cardiological, and anaesthetic colleagues.
